# Global gene expression in *Escherichia coli*, isolated from the diseased ocular surface of the human eye with a potential to form biofilm

**DOI:** 10.1186/s13099-017-0164-2

**Published:** 2017-04-03

**Authors:** Konduri Ranjith, Kotakonda Arunasri, Gundlapally Sathyanarayana Reddy, HariKrishna Adicherla, Savitri Sharma, Sisinthy Shivaji

**Affiliations:** 1 Jhaveri Microbiology Centre, Brien Holden Eye Research Centre, L V Prasad Eye Institute, Kallam Anji Reddy campus, Hyderabad, 500007 India; 2 CSIR-Centre for Cellular and Molecular Biology, Hyderabad, India; 3 Research Scholar, Manipal University, Manipal, Karnataka 576104 India

**Keywords:** Ocular *E. coli*, Biofilm, Antibiotic susceptibility, DNA microarray, Differential expression of genes

## Abstract

**Background:**

*Escherichia coli,* the gastrointestinal commensal, is also known to cause ocular infections such as conjunctivitis, keratitis and endophthalmitis. These infections are normally resolved by topical application of an appropriate antibiotic. But, at times these *E. coli* are resistant to the antibiotic and this could be due to formation of a biofilm. In this study ocular *E. coli* from patients with conjunctivitis, keratitis or endophthalmitis were screened for their antibiotic susceptibility and biofilm formation potential. In addition DNA-microarray analysis was done to identify genes that are involved in biofilm formation and antibiotic resistance.

**Results:**

Out of 12 ocular *E. coli* isolated from patients ten isolates were resistant to one or more of the nine antibiotics tested and majority of the isolates were positive for biofilm formation. In *E. coli* L-1216/2010, the best biofilm forming isolate, biofilm formation was confirmed by scanning electron microscopy. Confocal laser scanning microscopic studies indicated that the thickness of the biofilm increased up to 72 h of growth. Further, in the biofilm phase, *E. coli* L-1216/2010 was 100 times more resistant to the eight antibiotics tested compared to planktonic phase. DNA microarray analysis indicated that in biofilm forming *E. coli* L-1216/2010 genes encoding biofilm formation such as cell adhesion genes, LPS production genes, genes required for biofilm architecture and extracellular matrix remodeling and genes encoding for proteins that are integral to the cell membrane and those that influence antigen presentation are up regulated during biofilm formation. In addition genes that confer antimicrobial resistance such as genes encoding antimicrobial efflux (*mdt*M and *cyc*A), virulence (*ins*Q, *yjg*K), toxin production (*sat, yjg*K, *chp*S, *chp*B and *ygj*N), transport of amino-acids and other metabolites (*cbr*B, *cbr*C, *his*I and *mgl*B) are also up regulated. These genes could serve as potential targets for developing strategies for hacking biofilms and overcoming antibiotic resistance.

**Conclusions:**

This is the first study on global gene expression in antibiotic resistant ocular *E. coli* with a potential to form biofilm. Using native ocular isolates for antibiotic susceptibility testing, for biofilm formation and global gene expression is relevant and more acceptable than using type strains or non clinical strains which do not necessarily mimic the native isolate.

**Electronic supplementary material:**

The online version of this article (doi:10.1186/s13099-017-0164-2) contains supplementary material, which is available to authorized users.

## Background

*Escherichia coli*, is a coliform bacterium that occurs as a commensal in the gut of humans and other warm blooded mammals [1] and exhibits high degree of genotypic and phenotypic diversity. A few of them are pathogenic [2, 3] and include the verotoxigenic (VTEC), entero-haemorrhagic (EHEC, a subclass of the VTEC class), entero-invasive (EIEC) and uro-pathogenic/extra-intestinal pathogenic (UPEC/ExPEC) classes and are harmful to their hosts [3, 4]. *E. coli* also inhabits different environmental niches viz., soil, water, sediment, food, abiotic and biotic surfaces [5] like the surface of the eye and the inner surface of the eye lids. But, *E. coli* is not the only bacterium on the ocular surface and includes several other Gram-negative bacteria (*Neisseria* spp., *Pseudomonas aeruginosa* and *Haemophilus influenzae*) [6, 7], Gram-positive bacteria (*Staphylococcus epidermidis*, *S. aureus, Corynebacterium* spp., *Streptococci* spp., and *Propionibacterium acnes*), fungi (*Fusarium solani, Cladosporium sphaerospermum, Acremonium implicatum, Candida albicans* and *Aspergillus fumigatus*) [8–10] and viruses. More recently using non-cultivable molecular techniques based on 16S rRNA gene sequencing a greater bacterial diversity has been observed associated with the eye [11–14]. In all these studies the diversity of bacteria was very different and genera *Staphylococcus, Corynebacterium, Propionibacterium* and *Streptococcus* were the only common genera. Thus based on these studies it is difficult to conclude as to what constitutes the ocular surface microbiota (or a core microbiome) or whether the microbiota are only transiently present [15].

Characterising the ocular microbiome is important because following trauma or under immuno-compromised conditions these ocular microbiota may cause infection of the eye (such as keratitis, endophthalmitis, orbital cellulitis etc.) often leading to loss of vision. But, ocular infections such as conjunctivitits and keratitis may also originate from dirty fingers and contaminated contact lenses. Normally the infection is resolved following treatment with antifungal/antibacterial agents. However, over the years many of these organisms have become resistant to drugs. In preliminary studies carried out at the Jhaveri microbiology centre at L V Prasad Eye Institute (a tertiary eye care center), Hyderabad, India, several corneal pathogens were observed to be tolerant to one or more ocular antibiotics thus implying the emergence of tolerant strains [16]. Resistance to drugs may be linked to biofilm formation. Bacteria in a biofilm exhibit increased resistance to antibiotics due to binding of the antibiotics to the extracellular polymeric substances (EPS), due to production of enzymes that inactivate antibiotics, due to nutrient and oxygen limitation, due to increase in the efficiency of efflux pumps and due to up regulation of drug resistance-associated genes [17, 18].

Bacteria have been reported to form biofilm on contact lenses, intraocular lenses, lid implants, orbit implants, socket implants, scleral buckles and suture material [19, 20]. *P. aeruginosa*, *S. epidermidis*, *Streptococcus* spp. and *Enterobacter* have been identified to be a part of the biofilm [18]. Katiyar et al. [21] demonstrated that 85% of the isolates from intraocular lenses represented by *P. aeruginosa*, *Staphylococcus aureus*, *S. epidermidis*, *Micrococcus luteus*, *S. marcescens*, *Neisseria* spp., *Moraxella* spp*., Bacillus* spp., *E. coli*, *Proteus mirabilis*, *Enterobacter agglomerans* and *Klebsiella* spp., exhibited the potential to form biofilms and were resistant to antibiotics.

Information on genes that are differentially regulated during biofilm formation and correlating the results to increased resistance to antimicrobials have not been studied in ocular bacteria. With this in view, in the present study ocular isolates of *E. coli* which are known to cause about 1.1% of the total ocular infections [22] such as keratitis [23], endophthalmitis [24, 25], conjunctivitis [6], pan-ophthalmitis [26] and eyelid abscess [27], were used as model systems to investigate antibiotic susceptibility and biofilm formation potential. In addition global gene expression was studied by DNA-microarray analysis to identify genes that are involved in biofilm formation and antibiotic resistance. This approach using the native ocular isolates for global gene expression is relevant considering that studies have indicated that use of type strains (or non clinical strains) may not be the right approach to understand antibiotic resistance in a biofilm forming microorganism [28, 29]. Since the type strains of the same species need not necessarily mimic exactly the native isolate. The results of this study would unravel the identity of genes that are differentially regulated during biofilm formation and identify differentially regulated genes related to increased resistance to antimicrobials.

## Methods

### Collection of samples and bacterial identification

The Jhaveri Microbiology Centre of the Prof. Brien Holden Eye Research Centre at the L V Prasad Eye Institute, Hyderabad, India receives over 4000 clinical samples per annum from patients with eye infections. These samples are processed for the detection of bacteria and fungi and subsequently the cultures are purified, identified and preserved in tryptone soya broth with 30% glycerol at −80 °C. This study used 12 *E. coli* isolates preserved between 2010 and 2014. All preserved isolates of bacteria were cultured on 5% sheep blood agar medium at 37 °C. A pure homogenous culture obtained after repeated streaking was then subjected to Vitek 2 compact (bioMérieux, France) analysis for identification of the bacterium. Tests were performed according to the manufacturer’s instructions and the cultures were identified with the database of the instrument.

### Biofilm detection

The potential to form a biofilm was assessed in 12 ocular isolates of *E. coli* using the microtiter/tissue culture plate (TCP) method [30]. In the TCP or crystal violet method a single colony of the bacterium was inoculated into 5 ml medium of brain–heart infusion (BHI) and tryptic soy broth (TSB) (HiMedia Laboratories, Secunderabad, India) separately and incubated overnight. The culture was then adjusted to 0.5 McFarland units, diluted 100-fold and 100 µl of the diluted inoculum was dispensed into a single well of a 96 well plate (Thermo Fisher Scientific, Nunclon™, Denmark) containing 100 µl of fresh medium. The plate was then incubated at 37 °C for 72 h. The broth was then discarded by inverting the plate and gently tapping it after which it was washed thrice with 200 µl of phosphate buffered saline (PBS, Sigma-Aldrich Corporation, St. Louis, MO, USA) and allowed to dry for 30 min. The bacteria in the biofilm adhering to the plate were then stained with 200 µl of 0.1% Crystal violet for 30 min and the Crystal violet associated with the cells was then extracted with 200 µl of ethanol and quantified spectrophotometrically at 595 nm [30]. Visually, wells without the inoculum (control) and wells with bacteria that did not possess the potential to form biofilm were white to pale blue in colour. But, wells inoculated with bacteria which have a potential to form a biofilm are moderate to dark blue in colour indicative of biofilm formation. Wells without cells inoculated served as the control and the OD was <0.1 at 595 nm and was deducted from the biofilm positive strains (OD > 0.3 at 595 nm) and the biofilm negative strains (OD < 0.3 at 595 nm).

The TCP method was used to ascertain the optimum temperature and pH required for biofilm formation using two different media. The 12 ocular *E. coli* isolates were either incubated at 30 or 37 °C in BHI or TSB and the pH of the medium was adjusted to 5, 6, 7, 8 or 9 and incubated for 72 h. Following crystal violet staining the biofilm was quantified as above by recording the absorbance at 595 nm.

### Visualization of biofilm by scanning-electron microscopy (SEM) and confocal laser scanning microscope (CLSM)

A single colony of *E. coli* L-1216/2010 was incubated overnight at 37 °C in BHI or TSB. The culture was then adjusted to 3 McFarland with the medium and then 20 µl of 3 McFarland culture was made up to 200 µl using the same medium and transferred on to a sterile glass cover slip (10 × 10 mm) and placed in the 12 well plate (Thermo Fisher Scientific, Nunclon™, Denmark) and incubated for different time intervals (24, 48 and 72 h.) at 37 °C. After incubation, the adhering biofilm was washed, fixed with 2.5% glutaraldehyde solution for 2 h, washed thrice with distilled water and dehydrated through a graded series of ethanol (10, 25, 50, 70, 90, and 100%) for 20 min in each grade. Finally, the biofilms were air dried at 37 ± 1 °C for 24 h. Prior to visualisation of the biofilm using a SEM (HITACHI-Model S-3400N, Japan) the biofilms were metalized by gold sputtering for 45 s in a High Vacuum Evaporator (SC7620 PALARON Sputter Coater).

*Escherichia coli* biofilms of strain L-1216/2010 were also visualized by CLSM. The biofilms were grown for 24, 48 and 72 h as above, washed twice gently with autoclaved water and the adhering biofilm fixed with 4% formaldehyde (Himedia-Secunderabad, India) for 45 min. The fixed biofilms were then washed twice with autoclaved water and stained with 200 µl of 1.67 µM Syto^®^9 nuclear fluorescent dye (Thermo Fisher Scientific, USA) for 30 min. The stained biofilms were washed again with autoclaved water and mounted on a glass slide using mounting media (Thermo Fisher Scientific, USA). Confocal images were taken using Zeiss confocal laser scanning microscope (LSM 510). Argon Laser was excited at 450–490 nm, a 40× objective was used set at Zoom 2.

### Antibiotic susceptibility of ocular *E. coli*

The susceptibility of the 12 clinical isolates of *E. coli* to different antibiotics as listed in Table 1 was determined on Mueller Hinton agar medium (MHA-Himedia) by Kirby Bauer disc diffusion method as per CLSI 2012 guidelines [31] to determine the susceptibility of the isolates to different antibiotics. In addition the minimum inhibitory concentration (MIC) of the antibiotic required to inhibit the growth of the 12 isolates was determined by the micro-dilution method. For this purpose, overnight grown culture from the BHI medium was adjusted to 0.5 McFarland and 200 µl was transferred into each well of the 96 well plate and incubated at 37 °C for 16 h as described by The European Committee on Antimicrobial Susceptibility Testing (EUCAST) in 2003 [32] in the presence of the antibiotic. At the end of the incubation period OD 595 nm was determined. Each antimicrobial concentration was tested thrice and the mean values are presented. All the antibiotics were obtained from commercial sources. The susceptibility of *E. coli* isolate L-1216/2010 was also determined after the formation of the biofilm. In this experiment, overnight grown culture from then BHI medium was adjusted to 0.5 McFarland and 200 µl was transferred into each well of the 96 well plate and incubated at 37 °C for 48 h for biofilm formation. Planktonic cells were discarded, bound cells washed with milliQ water and then the antibiotic (dissolved in BHI medium) of a specific concentration was added to the biofilm and incubated for 16 h. The capability of the bacterium to form biofilm was monitored in the absence and the presence of different antibiotics at different concentrations by the crystal violet method. The concentration of the antibiotic that inhibited the formation of the biofilm was determined. *E. coli* isolate L-1216/2010 in the planktonic phase (where in antibiotics were added after 24 h of growth) and non-biofilm forming *E. coli* (L-1339/2013) were used as controls for this experiment.

**Table 1:** Resistance profile of 12 ocular *E. coli* to nine antibiotics by the Kirby–Bauer disc diffusion method and their biofilm forming potential by and the tissue culture plate (TCP) method

S. no	*E. coli* strain	Ocular sample	Patient diagnosis	Resistance to antibiotics (µg/disc)	Biofilm production by TCP method ±
1	L-1339/2013	Conjunctival swab	Conjunctivitis	Ce, Cef, Ci, Ga, Ge, Mo, Of	−
2	L-1216/2010	Vitreous	Endophthalmitis	Ci	+
3	L-2561/2013	Vitreous	Endophthalmitis	None	+
4	L-1920/2011	Corneal scraping	Keratitis	Am, Ce, Cef, Ch, Ci, Ga, Ge, Mo, Of	+
5	L-3781/2010	Vitreous	Endophthalmitis	Ce	+
6	L-3484/2010	Vitreous	Endophthalmitis	None	−
7	L-1573/2013	Vitreous	Endophthalmitis	Ci, Mo, Of	+
8	L-494/2011	Vitreous	Endophthalmitis	Am, Ce, Cef, Ci, Ga, Ge, Mo, Of	+
9	L-223/2014	Corneal scraping	Keratitis	Ce, Cef, Ci, Ga, Mo, Of	+
10	L-304/2014	Corneal scraping	Keratitis	Ce, Cef	+
11	L-811/2014	Lacrimal sac	Dacryocystitis	Ce, Cef, Ci, Ga, Ge, Mo, Of	+
12	L-823/2014	Corneal scraping	Keratitis	Cef	+

The antibiotics used are (µg/disc): *Am* amikacin (30), *Ce* ceftazidime (30), *Cef* cefuroxime (30), *Ch* chloramphenicol (30), *Ci* ciprofloxacin (5), *Ga* gatifloxacin (5), *Ge* gentamicin (10), *Mo* moxifloxacin (5) and *Of* ofloxacin (5)

+, biofilm positive strains (black colonies); −, biofilm negative strains (red or pink colonies). biofilm positive strains (OD > 0.3 at 595 nm); −, biofilm negative strains (OD < 0.3) at 595 nm. The OD of the control wells was deducted in each case

### DNA microarray analysis

RNA was extracted from biofilm cells and non-biofilm cells incubated for 72 h at 37 °C in BHI broth. For biofilm cells, *E. coli* (L-1216/2010) cells were allowed to grow and attach to plastic petri plates for 72 h (100 mm diameter containing 15 ml medium). After 72 h the planktonic cells were removed and only cells that were attached to the petri plate were considered as the biofilm cells and scrapped from the petriplate and were collected in a Falcon tube. The non-biofilm *E. coli* L-1339/2013 was used as the control. Cells of *E. coli* L-1339/2013 were allowed to grow for 72 h, centrifuged at 10,000 rpm in a microcentifuge (Eppendorf, New York, USA) and the pellet was collected. The pellets of the biofilm cells and non-biofilm cells were suspended separately in 1 ml of ice cold phosphate buffer (pH 7.2) to which 3 ml of RNA*later* ^®^ (Invitrogen BioServices India Pvt. Ltd, Bangalore 560 066, India) was added and the suspensions incubated for 30 min. Collection of cells to the addition of RNA*later* ^®^ was completed within 15 min. Extraction of total RNA, cDNA synthesis and DNA microarray analysis of all the six samples was done as described in our previous paper [33]. Briefly for this, the total RNA from the samples was extracted using Qiagen RNeasy mini-prep kit. This is followed by cDNA synthesis from the RNA (6 μg) by reverse transcription process using the first strand cDNA synthesis kit (Invitrogen Bioservices India Pvt. Ltd., Bangalore). Then the cDNA was fragmented with DNAse 1 (Promega Corporation, Madison, USA) and then labelled with biotin at the 3′ end using the labelling reagent from Affymetrix (CA, USA) and Terminal transferase enzyme (Promega). *E. coli* Genome 2.0 gene chip arrays were used for the DNA microarray study (Affymetrix CA, USA). The chip contained the complete genome of four *E. coli* strains (viz., non-pathogenic *E. coli* K12 MG1655, uropathogenic *E. coli* strain CFT073 and enterohemorrhagic *E. coli* O157:H7 strains EDL 933 and Sakai). The gene chip consists of approximately 10,000 probe sets for the 20,366 genes of all the four strains of *E. coli*. Three biological replicates were used for each condition of the experiment. The fragmented and labeled cDNA of *E. coli* cells from each biological replicate were processed independently and hybridized with DNA microarray chip. Thus for each condition of the experiment three DNA microarray chips were used. Microarray chips were then scanned using Affymetrix 428 Array Scanner and GCOS software to obtain images of the chips and further processed to get the florescent intensity of the probe sets. The fluorescent intensities were generated for the hybridized probes for each of the DNA microarray chips separately and analysed. The fluorescent intensity images were then normalized for background correction and data was analyzed using Gene Spring 12.5 software. To identify the significantly differentially regulated genes we have normalized the data of all biological replicates of both biofilm and non-biofilm *E. coli* cells by using PLIER (probe logarithmic intensity error estimation) followed by statistical analysis such as unpaired T test and P value calculation. Genes that exhibited ≥2.0-fold increase or decrease (biofilm cells versus non-biofilm cells) in expression and P ≤0.05 were considered as significantly differentially regulated genes. The microarray data was submitted to Gene Expression Omnibus (GEO) web deposit of National Centre for Biotechnology Information (NCBI, Maryland, USA) with an accession number GSE77872.

### Annotation of the genes

The differentially regulated genes were classified based on their function using Clusters of Orthologous Groups (COG), a software annotation pipeline associated with the Prokaryotic Genome Analysis Tool (PGAT) [34]. The COG protein database was generated by comparing predicted and known proteins in all completely sequenced microbial genomes to infer sets of orthologs. Various sets of differentially expressed genes based on their functionality were analysed for their interactions using GeneMANIA cytoscape plugin network analysis web tool [35]. Using GeneMANIA we performed all the possible default network interactions available for *E. coli* viz., co-expression, genetic interactions, protein interactions and other interactions. When the gene list exceeded more than 100 Cytoscape plugin was employed to derive the networks.

### Validation of microarray data by real time PCR (RT-PCR)

Expression of the genes *ydc*T*, ECS1633, yjc*Q*, omp*C*, per*M, *waa*L*, foc*A*, fli*C and *opr*R was validated by RT-PCR following the protocol described in our previous paper [33]. The RT-PCR reactions were performed in triplicate. Relative expression of genes was calculated by ΔΔC_T_ method. Expression of 16S rRNA gene was used as an internal standard. All values reported represent the mean of three independent experiments.

## Results

### Ocular isolates of *E. coli* and antibiotic susceptibility

Twelve *E. coli* isolates used in this study were isolated from keratitis (corneal scrapings), conjunctivitis (conjunctival swabs), endophthalmitis (vitreous fluid) or dacryocystitis (lacrimal sac pus) patients visiting the L. V. Prasad Eye Institute (LVPEI), Hyderabad, India, between the year 2010 and 2014 (Table 1; Additional file 1: Table S1). The demographic profile of the patients along with some clinical details is shown in the Additional file 1: Table S1. The patients were diagnosed and treated as per the institutional protocol. Visual acuity at presentation and at last follow up is also given in the Additional file 1: Table S1.

The antibiotic susceptibility results of the 12 ocular *E. coli* isolates (Table 1) indicated that except two isolates (L-3484/2010 and L-2561/2013) from the vitreous which were susceptible to all the antibiotics tested the remaining ten isolates were resistant to at least one or more antibiotics. Isolate (L-1920/2011) from corneal scrapings was the only isolate that showed resistance to all the antibiotics.

### Biofilm potential of ocular isolates of *E. coli*

In the microtiter/tissue culture plate (TCP) method ten isolates were positive for biofilm formation and showed an OD >0.3 at 595 nm compared to the negative strains whose OD was consistently <0.3 at 595 nm (Table 1; Additional file 2: Figure S1). The best biofilm forming isolate L-1216/2010 by the TCP method had an OD of 3.4 after 72 h of growth. The optimum conditions for biofilm formation as determined by the TCP method for all the isolates was 37 °C at pH 7.3 when grown on BHI or TSB medium and incubated for 72 h. Growth and biofilm formation in *E. coli* strains was repeated three times under optimum conditions. All future experiments related to antibiotic susceptibility of the biofilm and the genes that are differentially regulated were done with L-1216/2010 which formed a biofilm and L-1339/2013 which did not form a biofilm as the control under the above optimum conditions.

**Fig. 1:**
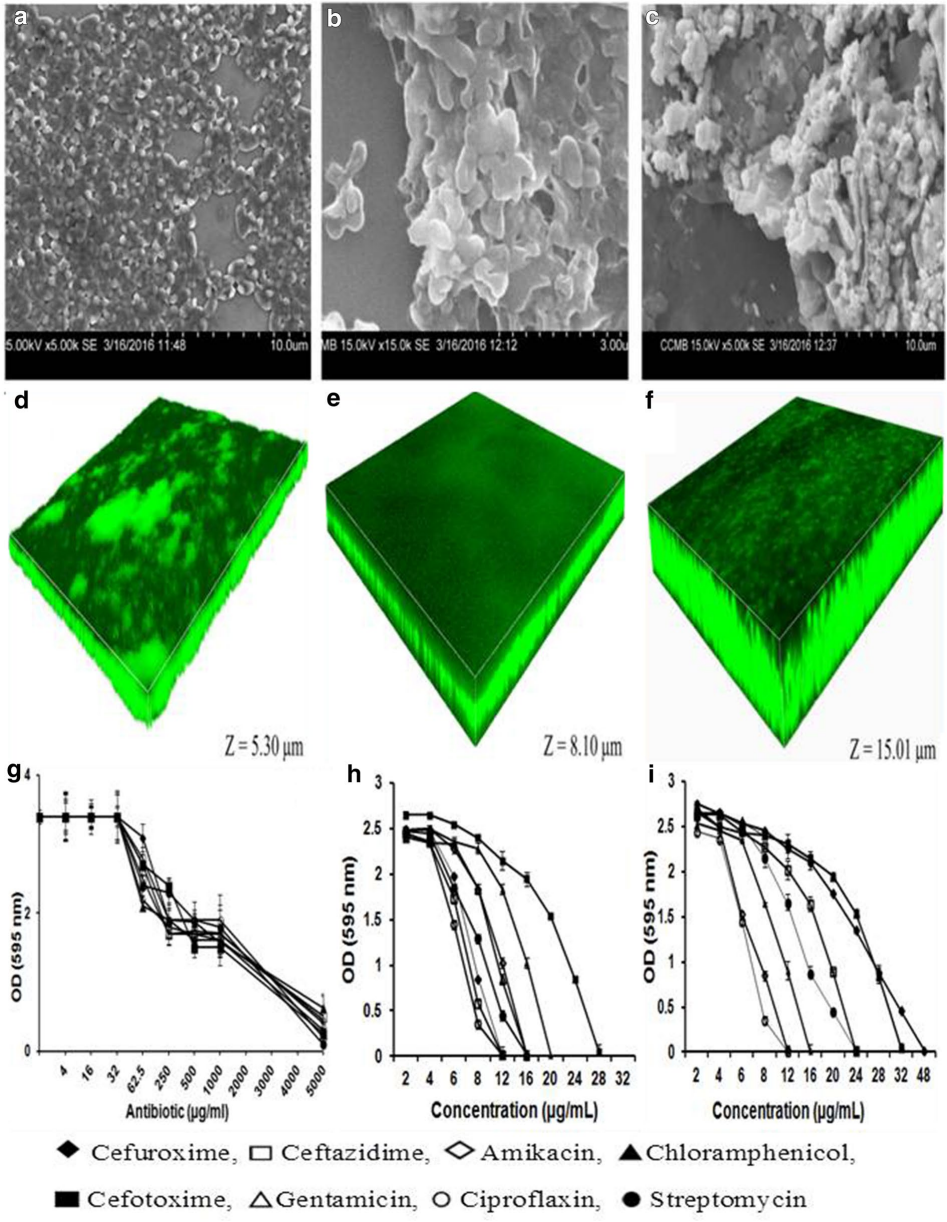
Biofilm forming potential in ocular *E. coli* L-1216/2010 as monitored by scanning electron microscopy after 24 (**a**), 48 (**b**) and 72 (**c**) h of biofilm growth and by confocal scanning laser microscopy after 24 (**d**), 48 (**e**) and 72 (**f**) h of biofilm growth. In **d**–**f**, the biofilm was stained with Syto9. The *Z axis* indicates the thickness of the biofilm which is 5.30, 8.10 and 15.01 μm after 24, 48 and 72 h of biofilm growth. **g** Represents the susceptibility of ocular *E. coli* L-1216/2010 in the biofilm phase, **h** represents the susceptibility of ocular *E. coli* L-1216/2010 in the planktonic phase and **i** represents the susceptibility of non-biofilm forming *E. coli* L-1339/2013 to different concentrations of antibiotics

Biofilm of ocular *E. coli* L-1216/2010 was also visualised by scanning electron microscopy (HITACHI-Model S-3400 N, Japan) (Fig. 1a–c). The scanning electronic microscopic images revealed that at 24 h cells were attached to the substratum and were evenly spread and the morphology of the cells was discernible. By 48 h the cells were entangled in the EPS and by 72 h a luxuriant biofilm was formed and the cells were totally covered within the biofilm and individual cells were not clearly visible. Biofilm formation in ocular *E. coli* L-1216/2010 was also confirmed by using a Zeiss confocal laser scanning microscope. The results clearly indicated an increase in the thickness of the biofilm with growth. The biofilm increased in thickness from 5.30 μm after 24 h to 15.01 μm at 72 h of growth (Fig. 1d–f).

The antibiotic susceptibility of biofilm of ocular *E. coli* L-1216/2010 was determined after allowing the cells to form a biofilm for 48 h. Three biological replicates of ocular *E. coli* L-1216/2010 were used for this process. The results indicated that *E. coli* L-1216/2010 in the biofilm phase required >5 mg/ml antibiotic to exhibit total susceptibility to the tested antibiotics where as planktonic cells of *E. coli* 1216/2010 (Fig. 1h) and *E. coli* L-1339/2013 (Fig. 1i) and the remaining ten strains of ocular *E. coli* were less tolerant and exhibited no growth at concentrations ranging from 0.005 to >0.064 mg/ml depending on the antibiotic tested (Fig. 1g–i; Table 2).

**Table 2:** Minimum inhibitory concentrations of antibiotics against 12 ocular *E. coli* isolates

*E. coli* strain	Sample	Amikacin	Ceftazidime	Cefuroxime	Chloramphenicol	Ciprofloxacin	Gentamicin	Streptomycin	Cefotaxime
		Antibiotics MIC (µg/ml)							
L-1339/2013	Conjunctival swab	12	24	48	16	12	32	24	32
L-1216/2013	Vitreous	16	12	28	20	12	12	16	16
L-2561/2013	Vitreous	6	2	4	4	0.5	2	12	1
L-1920/2011	Corneal scraping	64	16	32	32	12	16	8	12
L-3781/2010	Vitreous	12	16	8	6	0.5	2	16	16
L-3484/2010	Vitreous	12	2	4	6	1	2	8	2
L-1573/2010	Vitreous	6	4	8	4	4	4	8	1
L-494/2011	Vitreous	64	16	32	6	8	16	12	12
L-223/2014	Corneal scraping	12	24	48	8	4	4	16	32
L-304/2014	Corneal scraping	6	24	32	6	0.5	2	8	1
L-811/2014	Lacrimal sac	6	16	32	4	4	32	12	32
L-823/2014	Corneal scraping	6	2	24	2	0.5	4	6	1

### Expression of genes in *E. coli* biofilm forming cells

**Fig. 2:**
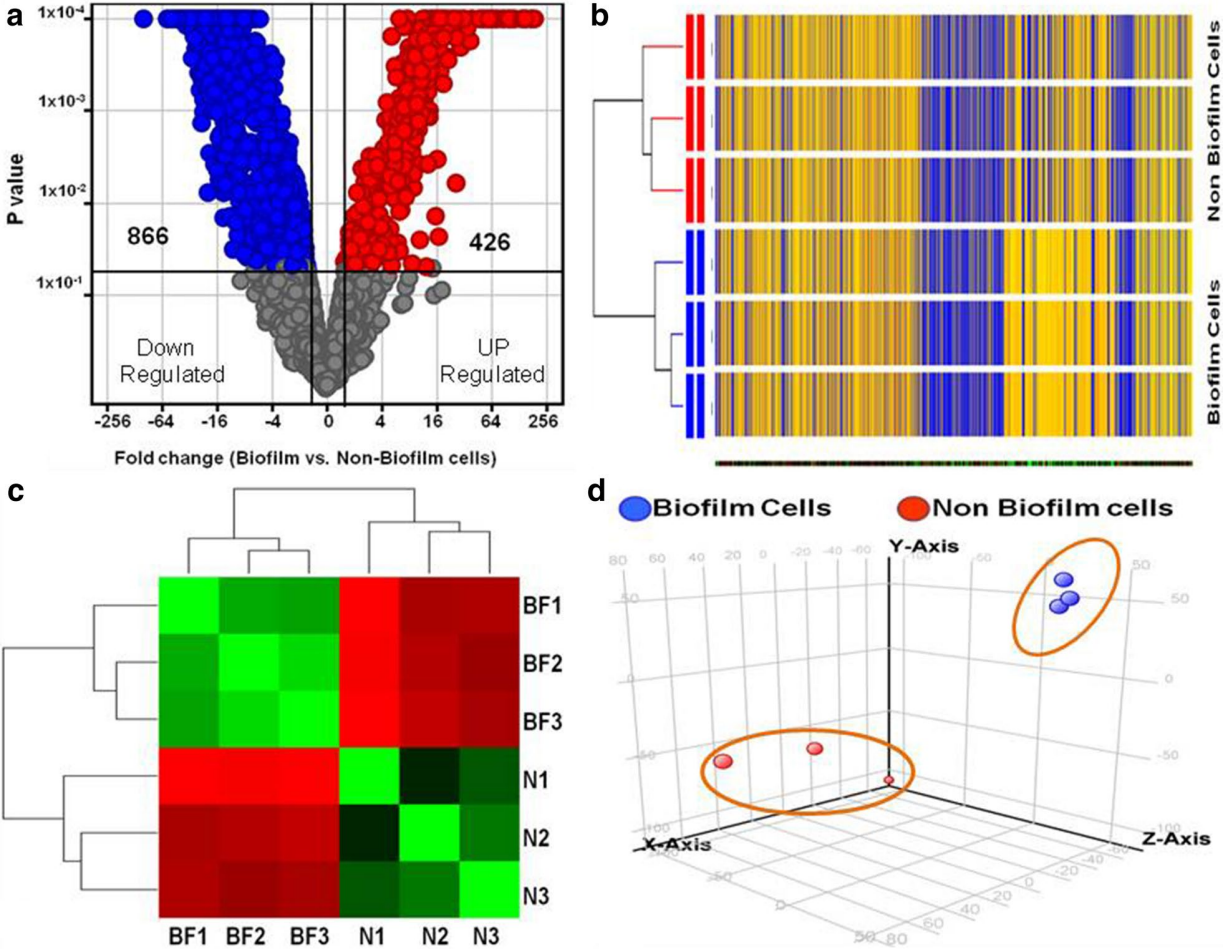
Differential gene expression in f *E. coli* (L-1216/2010) with potential to form biofilm versus non-biofilm forming cells of *E. coli* (L-1339/2013) grown for 72 h. In **a**, in the volcano plots genes that are represented on the *right side* of the volcano-axis are up regulated and those that are on *left side* of the axis are down regulated. In **b**, cluster analysis of biofilm forming *E. coli* (L-1216/2010) with non-biofilm forming cells of *E. coli* (L-1339/2013). In **c**, heat map analysis shows that the biofilm cells (BF1–BF3) are less related to non-biofilm cells (N1–N3) of *E. coli.* Principal component analysis (**d**)

Analysis of the microarray data indicated that in the biofilm forming ocular *E. coli* (L-1216/2010) cells after 72 h of biofilm formation, hundreds of genes were significantly differentially expressed with a fold change >2 (P < 0.05) compared to the non biofilm cells of *E. coli* L-1339/2013 (Fig. 2a). It was noted that 1292 (426 up and 866 down regulated) genes were significantly differentially regulated in the biofilm forming *E. coli* (L-1216/2010) cells compared to the non-biofilm forming *E. coli* L-1339/2013 cells. The cluster analysis (Fig. 2b) and heat maps (Fig. 2c) of the microarray data generated using mRNA of three biological replicates of biofilm cells of *E. coli* L-1339/2013 (BF1, BF2, BF3) and non biofilm forming cells from three biological replicates of *E. coli* L-1339/2013 (N1, N2, N3) also clearly showed that the biofilm cells were different from the non-biofilm cells (Fig. 2b, c). The Principal Component analysis also confirmed that the biofilm cells are more closely related and are less related to the non-biofilm cells (Fig. 2d).

### Functional annotation of differentially expressed genes in *E. coli* biofilm forming cells

Functional annotations and cluster categorization of the genes was done using DAVID, KEGG and COG analysis [33, 34]. Genes that could not be annotated and the duplicate genes were removed from the differentially expressed gene list of microarray data. This resulted in differential expression of a total of 1060 (385 upregulated and 675 downregulated) genes (Additional file 3: Table S2, Additional file 4: Table S3). Using COG all the genes could be categorized into four main categories viz., cellular processes and signaling, information storage and processing, metabolism and poorly characterized or unknown genes (Table 3). Each category included a number of sub-categories and the differentially regulated genes (up and down regulated) were compared at the sub-category level with data obtained in this study and three earlier studies (Table 3).

**Table 3:** Comparison of the differentially regulated genes in this study and three earlier studies based on COG sub-categories

Functional group classification based on COG	Beloin et al. [40]		Domka et al. [42]		Hancock et al. [43]		This study	
*E. coli* strain	*E. coli* K-12		*E. coli* K-12		ABU *E. coli*		Ocular *E. coli*	
Regulation	Up	Down	Up	Down	Up	Down	Up	Down
Cellular processes and signaling								
M. Cell wall/membrane/envelope biogenesis	19	9	9	8	4	25	14	14
D. Cell cycle control, mitosis and meiosis	2	1	0	0	3	6	14	14
N. Cell motility	NA	NA	32	1	1	0	7	19
O. Post-translational modification, protein turnover, and chaperones	13	13	13	7	25	56	8	11
T. Signal transduction mechanisms	5	0	11	3	12	2	7	5
U. Intracellular trafficking, secretion, and vesicular transport	NA	NA	28	1	2	17	8	19
V. Defense mechanisms	NA	NA	4	0	1	0	1	0
Sub-total	39	23	97	20	48	106	59	82
Information storage and processing								
A. Transcription, RNA processing and modification	11	5	17	7	44	8	12	6
L. Replication, recombination and repair	10	7	5	1	17	14	16	10
Sub-total	21	12	22	8	61	22	28	16
Metabolism								
C. Energy production and conversation	23	9	23	16	12	34	12	18
E. Amino acid transport and metabolism	9	22	20	15	5	36	20	17
F. Nucleotide transport and metabolism	0	3	8	1	6	8	5	5
G. Carbohydrate transport and metabolism	16	22	27	5	13	23	26	12
H. Coenzyme transport and metabolism	3	5	6	1	9	14	5	7
I. Lipid transport and metabolism	7	0	5	3	3	8	2	5
P. Inorganic ion transport and metabolism	12	6	24	9	14	7	4	8
Q. Secondary metabolites biosynthesis, transport and catabolism	2	1	5	0	2	7	5	2
Sub-total	72	68	118	50	64	137	79	74
Unknown and general prediction only	121	86	53	6	57	23	24	29
Total	253	189	290	84	230	288	190	201

The figures in each column indicate the number of genes up or down regulated in each category. Comparison between the four studies in terms of genes up or down regulated are not anticipated to be identical but the trends may be similar. For instance the number of genes up or down regulated when ABU *E. coli* is compared with ocular *E. coli* is similar for category V, L, F and I. Such comparisons could also be done between ocular *E. coli* and the other two strains of *E. coli*

### Validation of DNA microarray results

**Fig. 3:**
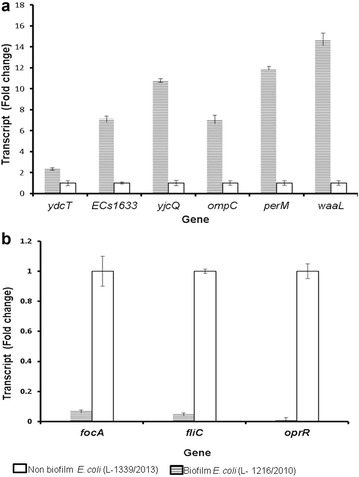
Real-time PCR validation of the expression *of* genes in biofilm (*closed box*) and non-biofilm cells (*hatched box*) of *E. coli* (L-1216/2010) and *E. coli* (L-1339/2013) (*open box*) respectively. **a** Relative expression of up-regulated genes and **b** Relative expression of down-regulated gene

Expression of few of the genes that were differentially regulated in the sessile biofilm cells was validated by RT-PCR. In accordance with DNA microarray results genes *ydc*T*, ECS1633, yjc*Q*, omp*C*, per*M and *waa*L showed increased expression (P < 0.05) whereas genes *foc*A*, fli*C and *opr*R showed decreased expression (P < 0.05) in *E. coli* (L-1216/2010) with potential to form biofilm compared with *E. coli* (L-1339/2013) that does not form a biofilm (Fig. 3a, b).

### Genemania network analysis

**Fig. 4:**
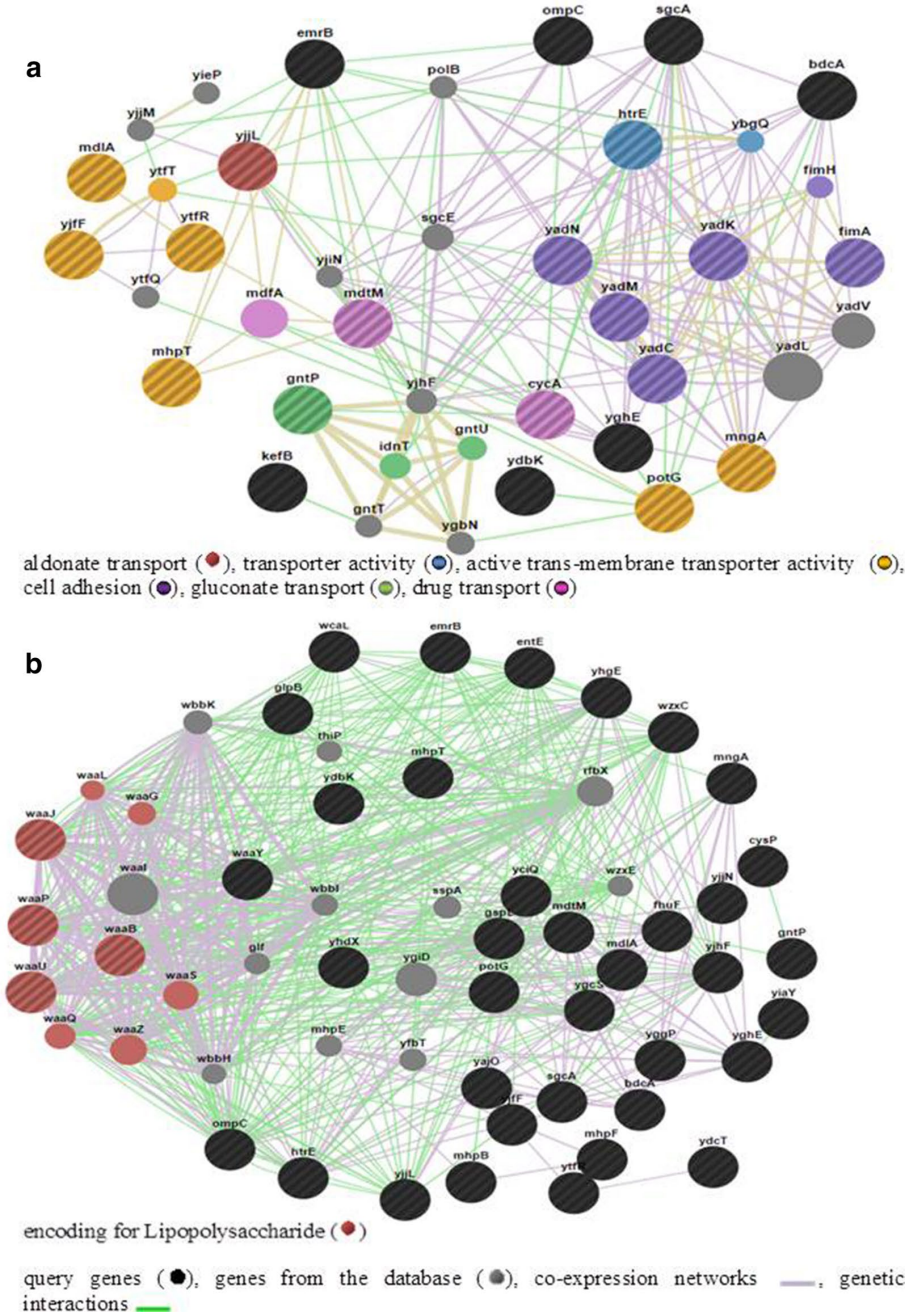
GeneMANIA network analysis of cell adhesion and transport genes. **a** Represents the interaction of genes at nodes encoding for transport activity with the cell adhesion genes. **b** Represents interaction of genes encoding for lipopolysaccharide

Following functional annotation and cluster categorization of the up regulated genes using DAVID, the up regulated genes in biofilm forming *E. coli* (L-1216/2010) cells were networked using GeneMANIA. GeneMANIA deduces networks by integrating publicly available genomic and proteomic data of 33 previous studies. GeneMANIA network analysis predicts that cell adhesion genes (*fim*A, *yad*K, *yad*N, *yad*M and *yad*C) were co-expressed along with genes encoding drug transport (*mdt*M and *cyc*A), aldonate transport (*yjj*L), transporter activity (*htr*E), active transmembrane transporter activity (*pot*G, *mng*A) and gluconate transport (*gnt*P) (Fig. 4a). Network analysis also revealed that *emr*B interacts with proteins encoded by *mdt*M and *mdf*A involved in multidrug resistance (Fig. 4a) thus accounting for the observed up regulation of *mdl*A and *mdt*M. Gene *emr*B also shared genetic interaction with several genes involved in aldonate and gluconate transport (Fig. 4a). Up regulated genes in the biofilm forming cells belonging to lipopolysaccharide biosynthetic process (viz., *waa*J, *waa*P, *waa*U and *waa*B) were co-expressed in the network analysis (Fig. 4b). Network analysis also indicated that several of the genes integral to cell membrane (*yii*X, *cbr*B, *cbr*C, *his*I) interact with one another (Additional file 5: Figure S2) and with genes encoding for putative fimbriae like proteins (*yad*C*, yad*L *and yad*M), outer membrane proteins (*htr*E), transcriptional regulators (*mng*R, *nha*R), DNAdamage repair proteins (*uvr*D), cytosine deaminase (*cod*A) and other genes. Network analysis also indicated that the hypothetical genes are interlinked to ABC transport protein genes, DNA replication, *rrna* genes and outer membrane genes and may thus be functionally useful for biofilm formation and virulence characteristic of the bacterium (Additional file 6: Figure S3).

## Discussion

Our results indicate that 10 out of the 12 ocular *E. coli* isolates from conjunctival swab, corneal scrapings, vitreous fluid or lacrimal sac of patients were resistant to at least one or more of the nine antibiotics tested. Out of these ten antibiotic tolerant ocular *E. coli*, majority of the isolates (8/10) were positive for biofilm formation by the TCP method thus implying a close association between antibiotic tolerance and biofilm formation [36, 37]. Surprisingly we also observed that *E. coli* L-2561/2013 from the vitreous fluid of Endophthalmitis patients was not resistant to any one of the antibiotics tested but possessed the ability to form biofilm. This is indeed interesting. A recent study also indicated that biofilm formation was independent of antibiotic resistance. For instance in *Acinetobacter baumannii* out of 63 strong biofilm makers 79.4% were non-multidrug-resistant strains and the authors observed a negative correlation between antibiotic resistance and biofilm forming capacity [38]. This observation was interpreted as indicating that biofilm improves the survival of bacteria in which resistance is low [38]. The study also indicated that ocular *E. coli* L-1216/2010, required >5 mg antibiotic for total susceptibility in the biofilm phase compared to microgram quantities that were required to inhibit the growth in the planktonic phase thus implying 100-fold increase in resistance in the biofilm phase. These observations confirm an earlier study in *Pseudomonas aeruginosa*, *E. coli*, coagulase-negative *Staphylococcus* etc. which had also indicated increased resistance to antibiotics in biofilm phase versus planktonic phase. This is the first study on antibiotic susceptibility of an ocular isolate of *E coli* in the biofilm phase to different antibiotics.

Differential gene expression in both pathogenic and non-pathogenic *E. coli* with the ability to form biofilm has been studied earlier (*E. coli* K12 and ABU *E. coli*) using various platforms and the results indicated that several genes were differentially regulated and the number varied from 233 to 815 genes representing about 5.4–19.07% of the total genes [39–43]. The primary aim of this study was to identify genes that are differentially expressed during biofilm formation and also demonstrate that during biofilm formation the genes that facilitate drug resistance are up regulated. Therefore we chose L-1216/2010 to represent biofilm forming *E. coli* since it was the best biofilm forming isolate and resistant to just only one antibiotic and as a control we chose L-1399/2013 which was resistant to seven antibiotics and did not form a biofilm. This approach would allow us to identify genes which are up-regulated and associated with biofilm formation and also genes which are involved in drug resistance over and above the drug resistant *E. coli* L-1399/2013 which does not form a biofilm.

In the present study, we observed that in biofilm forming ocular *E. coli* L-1216/2010 the number of genes that were significantly differentially regulated during biofilm phase were higher (385 and 675 genes respectively were up and down regulated) compared to the previous studies. Comparison of the differentially regulated genes in ocular *E. coli* with that of *E. coli* K12 and ABU *E. coli* revealed that differential regulation of genes of the sub-category U (intracellular trafficking, secretion, and vesicular transport), V (defence mechanisms) and L (Replication, recombination and repair) were similar in pathogenic ABU *E. coli* and ocular *E. coli* (Table 3). Further, ocular *E. coli* could be differentiated from *E. coli* K12, a laboratory strain, with respect to regulation of genes belonging to the sub-categories D (cell cycle control, mitosis and meiosis), N (cell motility), U (intracellular trafficking, secretion and vesicular transport) and V (defense mechanisms) (Table 3). In the subsequent part genes relevant to biofilm and virulence in antibiotic tolerant ocular *E. coli* L-1216/2010 would be discussed in comparison with the previously published data on non-pathogenic *E. coli* K12 and pathogenic ABU and uropathogenic *E. coli*.

### Genes involved in motility and adhesion

In motile *E. coli,* biofilm formation is correlated with its ability to swim [44, 45]. Thus it is not surprising that genes coding for a second flagellar system *fhi*A [46], minor tail protein *ECs1554*, *fim*A coding for type 1 fimbriae [39–41], *yad*L and *yad*M [47], Z5029 and Z1651 coding for putative fimbrial-like adhesin proteins are up regulated in biofilm forming ocular *E. coli* L-1216/2010. Among these genes *fhi*A and *ECs1554* are shown to be associated with biofilm formation in K-12 strains *of E. coli* [48, 49] and in one of the ABU strains VR50 [43]. But *fim* genes were not up regulated in expression in ABU strain 83972 [43] and in UPEC strain CFT073 [39]. Thus expression of genes encoding fimbriae and fimbriae related functions in *E. coli* appear to be strain-specific [50]. GeneMANIA network analysis also predicted a co-expression network interaction of *fim*A with that of fimbrial adhesion protein encoding genes *yad*L and *yad*M. Gene *bdc*A, a c-di-GMP-binding biofilm dispersal mediator protein was also up regulated in *E. coli* L-1216/2010 and co-expressed with genes encoding for cell adhesion (Fig. 4a). Several small molecules like Cyclic di-GMP (c-di-GMP) [51], acetyl phosphate (AcP) [52] and ppGpp promote biofilm formation. Biofilm formation in Gram negative bacterial cells is also facilitated by the adhesion of cells to the substratum which is conferred by the lipopolysaccharide (LPS) of the outer membrane [53]. In this study it is observed that genes coding for both the major components of LPS, namely hydrophobic lipid A moiety (*waa*B*, waa*P*, waa*J and *waa*R) and the phosphorylated core oligosaccharide (*waaU* and *waaY*) and those coding for the O antigen synthesis (*waa*L and *wzzB*) are up regulated. However, in contrast in pathogenic ABU strains of *E. coli,* genes encoding lipid A remain unchanged and genes involved in peptidoglycan biosynthesis were down-regulated [43]. LPS, in *E. coli* also acts as a virulence factor [54].

### Genes involved in biofilm architecture

Three exopolysaccharides, β-1,6-*N*-acetyl-d-glucosamine polymer (PGA) [55], colanic acid a negatively charged polymer of glucose, galactose, fucose and glucuronic acid and cellulose, have been detected in the biofilm matrix of *E. coli* and considered to be essential for normal biofilm architecture [56]. Concomitantly, in the present study *yhj*N coding for cellulose synthase regulator protein was up regulated where as in ABU strains of *E. coli* genes involved in cellulose synthesis (*bcs*ABZC and *bcs*EFGgenes) were down regulated [43]. Genes encoding colanic acid synthesis (*wca*L and *wca*M) were down regulated in the ocular *E. coli* and ABU strains [43] probably because expression of colanic acid inhibits the biofilm ability of *E. coli* [57]. Further in ocular *E. coli* L-1216/2010 colicin protecting conserved protein encoding genes *cbr*B and *cbr*C are up regulated by 11- and 288-folds respectively compared to the non-biofilm forming cells.

### Genes involved in drug transport and active transport

Genes encoding drug transport (*mdt*M and *cyc*A) and aldonate, active transport activity (*yjj*L, *htr*E, *pot*Gand *mng*A) (Fig. 4a) are differentially regulated in antibiotic tolerant ocular *E. coli* and may thus facilitate drug resistance [47]. In ocular *E. coli* in the biofilm phase gene *emr*B which confers resistance to cyanide *m*-chlorophenylhydrazone, tetrachlorosalicylanilide, organomercurials, nalidixic acid and thiolactomycin, genes *mdl*A and *mdt*M which encode multidrug drug efflux pumps [58] and the *sat* gene encoding the secreted auto-transporter toxin (Sat) are up regulated. *cyc*A that encodes for glycine, serine and alanine transport is predicted to genetically interact with gene *mdt*M and is 47-fold down regulated in biofilm forming *E. coli* cells. *cyc*A mutant strains of *E. coli* are known to exhibit significant resistance for d-cycloserine [59]. Thus down regulation of gene *cyc*A in biofilm forming *E. coli* cells significantly contributes to antibiotic tolerance. Apart from these efflux pumps, *omp*C encoding secretion of extracellular proteins, gene *htrE* encoding putative chaperone-usher fimbrial protein [47] and many genes encoding dehydrogenases (*mhpB, mhpF*, *yia*Y, *yaj*O/*ydb*K and *yjj*N) are up regulated. GeneMANIA network analysis of drug and active transport genes indicated that all these genes clustered through the genetic interaction networks and may facilitate drug resistance (Fig. 4a).

### Biofilm formation, virulence and other genes

Genes involved in pathogenicity, biofilm formation, resistance to antimicrobial compounds and virulence are related. Accordingly complete or partial upregulation of 22 genes (*c2418 to c2440*) in the pathogenicity island PAI IV_536_ in the UPEC strain 536 and in ABU strains VR50 and strain 83972 [43] was observed during biofilm formation. In addition, RfaH a virulence regulator, which regulates expression of several virulent genes in *E. coli* is significantly up regulated in ABU strains during biofilm phase [43]. None of the above genes was differentially regulated in ocular *E. coli* but up regulation of gene ECs3276 a virulence protein populating the cluster O80301 [60] was observed (Additional file 3: Table S2). Apart from this, upregulation of several genes encoding for toxin production and secretions (*sat, yjg*K, *chp*S, *chp*B and *ygj*N) was observed in ocular *E. coli* biofilm cells which is an important factor that governs the virulence in pathogenic bacterial cells. Virulence of ocular *E. coli* may also be related to up regulation of Lipid A moiety of LPS, a potent stimulator of the immune system and a trigger of intense inflammation in the host cells [61]. Many genes encoding for proteins that influence antigen presentation (*wzz*B *and waa*L), extracellular matrix remodeling (*yii*X), transport of aminoacids and other metabolites (*cbr*B, *cbr*C, *his*I and *mgl*B) are also up regulated in ocular *E. coli* in biofilm stage and may have a role to play in virulence. Costa et al. [61] have recently suggested that in *E. coli,* during biofilm formation on abiotic surfaces, cells are exposed to several DNA damaging agents against which the cells need to be protected. In the present study gene *ira*D, *uvr*D and *rec*C involved in DNA damage repair are up regulated and could be acting to ensure survival of cells faced with oxidative damage. Thus the virulence presentation in ocular *E. coli* biofilm cells may be an exclusive process. It was also observed that ocular *E. coli* in biofilm phase show up regulation of stationary phase response genes (*spe*B, *ybe*W, *hsc*C and *djl*C which encode for Agmatinase) and genes indicative of anaerobic conditions *(dsm*B, *glp*B and *nrd*D). However the stress response encoding genes up regulated in ABU strains of *E. coli* (such as *csp*G, *csp*H, *pph*A*, ibp*A, *ibp, sox*S*, hha* and *yfi*D) were not up regulated in ocular *E. coli* except *usp*C encoding a universal stress protein which was up regulated [39–41, 43].

Several other genes involved in metabolism, biosynthesis, transport, efflux pumps, cell membrane structure, DNA replication etc. are also differentially regulated in ocular *E. coli* (Additional file 3: Table S2) and may thus be related to biofilm formation, virulence or both. Beloin et al. [45] in a review stated that *E. coli* species have not yet revealed all the secrets that contribute to bacterial biofilm research and one of the reasons is attributed to the fact that many of the differentially expressed genes were categorized either as unknown or coding for hypothetical proteins. In antibiotic resistant ocular *E. coli* also a substantial number of the genes are not annotated.

Virulence in *E. coli* is also known to be associated with extrachromosomal elements such as plasmids, associated bacteriophages and pathogenicity islands [62, 63] and a similar up-regulation was observed in ocular biofilm forming *E. coli* L-1216/2010 (Additional file 7: Table S4). In addition it was observed that other genes such as those conferring protection against colicin action (*cbr*C) (Additional file 7: Table S4), ten toxin encoding prophage CP-933 related genes (as in *Escherichia coli* O157:H7 strain EDL933) [64], phage-related virulence protein encoding gene *ins*Q, biofilm modulator toxin encoding gene *yjg*K (Additional file 3: Table S2, Additional file 4: Table S3) have also been observed to be up regulated Thus it appears that the differential regulation of extrachromosomal elements may be implicated in the biofilm formation and antibiotic resistance in ocular *E. coli* L-1216/2010.

## Conclusions

We report the first global gene expression data of antibiotic resistant ocular biofilm forming *E. coli* cells and demonstrate that antibiotic tolerance of ocular biofilm forming *E. coli* L-1216/2010 is dependent on up regulation in expression of genes encoding drug transport, active transport, multi-drug efflux pumps and genes conferring tolerance to drugs. Simultaneously genes required for biofilm formation such as genes involved adhesion, LPS production and biofilm architecture are up regulated. This study also identifies hitherto unreported sets of oxidative stress protecting genes and extrachromosomal elements such as plasmids, associated bacteriophages and pathogenicity islands which are also up regulated during biofilm formation in ocular *E. coli*. It is envisaged that inhibition of the above up regulated genes by using inhibitors could serve as a strategy for preventing biofilm formation and overcoming drug resistance.

In subsequent studies we hope to validate a few of the genes for which functions have not been assigned by specific gene knock out studies by transposon mediated targeted mutagenesis as reported earlier by us [65–67]. Inhibition of up regulated genes either by using already known inhibitors or by designing new inhibitors would also be attempted as an alternative strategy.

## Additional files

**Additional file 1: Table S1.** Clinical profile of the patients included in the study.

**Additional file 2: Figure S1.** Biofilm forming potential in twelve ocular isolates of *E. coli* from Vitreous, Corneal scraping, Conjunctival swab and Lacrimal gland evaluated by tissue culture plate method. The dark blue sediment adhering to the bottom of the well is indicative of biofilm forming potential of the isolates. isolates from left to right are L-1339/2013, L-1216/2010, L-2561/2013, L-1920/2011, L-3781/2010, L-3484/2010, L-1573/2013, L-494/2011, L-223/2014, L-304/2014, L-811/2014 and L-823/2014 respectively. Except isolates L-1339/2013 and L-3484/2010 all the remaining isolates are positive for biofilm forming potential.

**Additional file 3: Table S2.** Up regulation of significantly differentially regulated genes in ocular *Escherichia coli* L-1216/2010 biofilm cells versus ocular *E. coli* L-1339/2013 cells which do not form a biofilm as the control.

**Additional file 4: Table S3.** Down regulation of significantly differentially regulated genes in ocular *Escherichia coli* L-1216/2010 biofilm cells versus ocular *E. coli* L-1339/2013 cells which do not form a biofilm as the control.

**Additional file 5: Figure S2.** Network analysis co-expression showing interaction of genes encoding for integral cell membrane proteins (*yii*X*, cbr*B, *cbr*C, *cyc*A, *his*L and *mgl*B) between themselves and with genes encoding for putative fimbriae like proteins (*yad*C*, yad*K*, yad*L *and yad*M), outer membrane proteins (*htr*E*),* transcriptional regulators (*mng*R*, nha*R), DNA damage repair (*uvr*D), cytosine deaminase (*cod*A) etc.

**Additional file 6: Figure S3.** Cytoscape network analysis showing interaction of genes encoding for hypothetical proteins.

**Additional file 7: Table S4.** Expression of up regulated extrachromosomal genes in ocular *Escherichia coli* L-1216/2013 compared to *Escherichia coli* K-12 substr. MG1655.

## Abbreviations

VTEC: verotoxigenic
EHEC: entero-haemorrhagic
EIEC: entero-invasive
ExPEC: extra-intestinal pathogenic
UPEC: uro-pathogenic
TCP: tissue culture plate
BHI: brain–heart infusion
TSB: tryptic soy broth
SEM: scanning-electron microscopy
CLSM: confocal laser scanning microscope
MHA: Mueller–Hinton agar medium
MIC: minimum inhibitory concentration
CLSI: Clinical and Laboratory Standards Institute
EUCAST: European Committee on Antimicrobial Susceptibility
PLIER: probe logarithmic intensity error estimation
GEO: Gene Expression Omnibus
COG: Clusters of Orthologous Groups
DAVID: Database for Annotation, Visualization and Integrated Discovery
KEGG: Kyoto Encyclopedia of Genes and Genomes
