# Supplementary figures and images for: Global gene expression in Escherichia coli, isolated from the diseased ocular surface of the human eye with a potential to form biofilm
Source: Gut Pathog. 2017 Apr 3;9:15. doi: 10.1186/s13099-017-0164-2 (PMC5379667; doi:10.1186/s13099-017-0164-2)

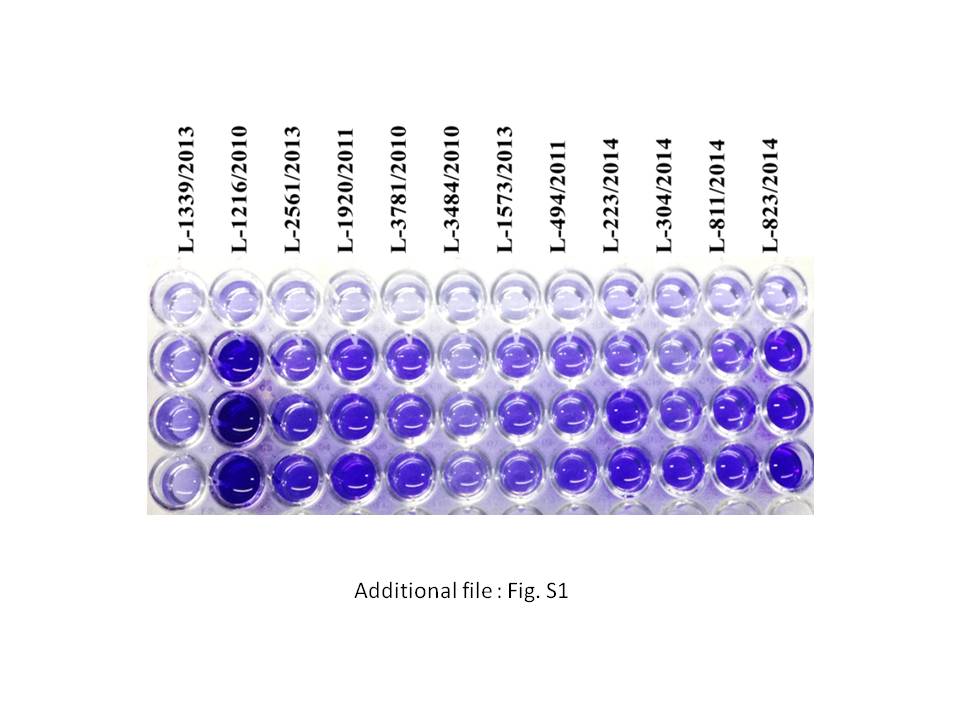

Supplement: Supplementary file 2 — Additional file 2: Figure S1. Biofilm forming potential in twelve ocular isolates of E. coli from Vitreous, Corneal scraping, Conjunctival swab and Lacrimal gland evaluated by tissue culture plate method. The dark blue sediment adhering to the bottom of the well is indicative of biofilm forming potential of the isolates. isolates from left to right are L-1339/2013, L-1216/2010, L-2561/2013, L-1920/2011, L-3781/2010, L-3484/2010, L-1573/2013, L-494/2011, L-223/2014, L-304/2014, L-811/2014 and L-823/2014 respectively. Except isolates L-1339/2013 and L-3484/2010 all the remaining isolates are positive for biofilm forming potential. [file 13099_2017_164_MOESM2_ESM.jpg]

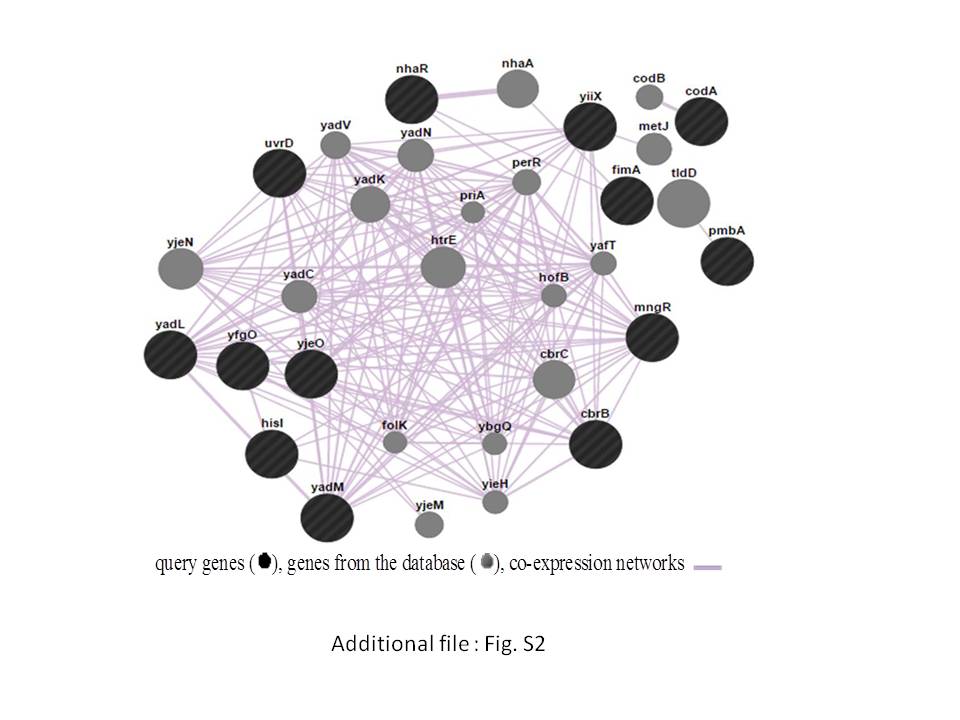

Supplement: Supplementary file 5 — Additional file 5: Figure S2. Network analysis co-expression showing interaction of genes encoding for integral cell membrane proteins (yiiX, cbrB, cbrC, cycA, hisL and mglB) between themselves and with genes encoding for putative fimbriae like proteins (yadC, yadK, yadL and yadM), outer membrane proteins (htrE), transcriptional regulators (mngR, nhaR), DNA damage repair (uvrD), cytosine deaminase (codA) etc. [file 13099_2017_164_MOESM5_ESM.jpg]

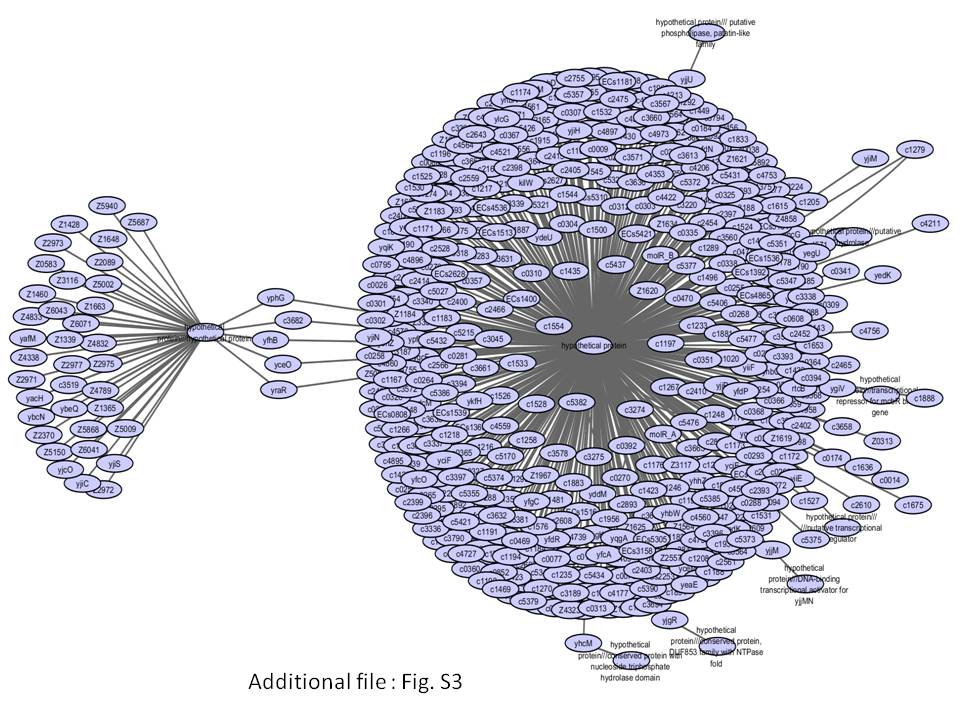

Supplement: Supplementary file 6 — Additional file 6: Figure S3. Cytoscape network analysis showing interaction of genes encoding for hypothetical proteins. [file 13099_2017_164_MOESM6_ESM.jpg]
